# Asymmetric tumor‐related alterations of network‐specific intrinsic functional connectivity in glioma patients

**DOI:** 10.1002/hbm.25140

**Published:** 2020-07-27

**Authors:** Kerstin Jütten, Verena Mainz, Daniel Delev, Siegfried Gauggel, Ferdinand Binkofski, Martin Wiesmann, Hans Clusmann, Chuh‐Hyoun Na

**Affiliations:** ^1^ Department of Neurosurgery RWTH Aachen University Aachen Germany; ^2^ Institute of Medical Psychology and Medical Sociology RWTH Aachen University Aachen Germany; ^3^ Division of Clinical Cognitive Sciences RWTH Aachen University Aachen Germany; ^4^ Department of Diagnostic and Interventional Neuroradiology RWTH Aachen University Aachen Germany

**Keywords:** default‐mode network, fronto‐parietal network, glioma, independent component analysis, neuropsychology, resting‐state functional connectivity

## Abstract

Resting‐state functional MRI (rs‐fMRI) allows mapping temporally coherent brain networks, and intra‐ and inter‐network alterations have been described in different diseases. This prospective study investigated hemispheric resting‐state functional connectivity (RSFC) differences in the default‐mode network (DMN) and fronto‐parietal network (FPN) between patients with left‐ and right‐hemispheric gliomas (LH PAT, RH PAT), addressing asymmetry effects the tumor might have on network‐specific intrinsic functional connectivity under consideration of the prognostically relevant isocitrate‐dehydrogenase (IDH) mutation status. Twenty‐seven patients (16 LH PAT, 12 IDH‐wildtype) and 27 healthy controls underwent anatomical and rs‐fMRI as well as neuropsychological assessment. Independent component analyses were performed to identify the DMN and FPN. Hemispheric DMN‐ and FPN‐RSFC were computed, compared across groups, and correlated with cognitive performance. Patient groups did not differ in tumor volume, grade or location. RH PAT showed higher contra‐tumoral DMN‐RSFC than controls and LH PAT. With regard to the FPN, contra‐tumoral RSFC was increased in both patient groups as compared to controls. Higher contra‐tumoral RSFC was associated with worse cognitive performance in patients, which, however, seemed to apply mainly to IDH‐wildtype patients. The benefit of RSFC alterations for cognitive performance varied depending on the affected hemisphere, cognitive demand, and seemed to be altered by IDH‐mutation status. At the time of study initiation, a clinical trial registration was not mandatory at our faculty, but it can be applied for if requested.

## Graphical abstract

This prospective study investigated hemispheric resting‐state functional connectivity (RSFC) differences in the default‐mode network (DMN) and fronto‐parietal network (FPN) between patients with left‐ and right‐hemispheric gliomas and healthy controls (LH PAT, RH PAT, HC), addressing asymmetry effects the tumor might have on network‐specific intrinsic functional connectivity under consideration of the prognostically relevant isocitrate‐dehydrogenase mutation status. Hemispheric RSFC in glioma patients varied depending on tumor hemisphere and investigated network, indicating a dissociation between tumor‐related DMN‐ and FPN‐RSFC changes. Hemispheric RSFC increases were not necessarily found to be beneficial for performance in terms of better performance measures in patients, but to vary depending on tumor side and cognitive demand, which seemed to be further altered by IDH‐mutation status.

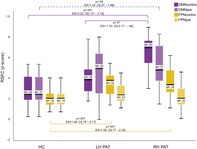

## 1 INTRODUCTION

Patients with gliomas often reveal cognitive deficits already at the time of diagnosis (Habets et al., 2014; Van Kessel, Baumfalk, van Zandvoort, Robe, & Snijders, 2017). While patients with low‐grade gliomas are initially less affected, patients with high‐grade gliomas often show more pronounced cognitive deficits. Correspondingly, the (presurgical) cognitive status has been shown to be strongly related to patients' survival (Meyers, Hess, Yung, & Levin, 2000; Van Kessel et al., 2019), emphasizing the significance of a multidimensional assessment of cognitive functions for patient care and treatment. As the preservation of cognitive functions is of paramount importance with regard to the social and vocational integration and patients' quality of life, it should play a decisive role in treatment planning and surgical intervention (Klein, Duffau, & De Witt Hamer, 2012). In this regard, mapping the organization of the underlying functional networks can be useful, as increasing evidence underlines that cognitive deficits in tumor patients exceed local tumor effects and rather depend on spatially distributed neural networks (Bullmore & Sporns, 2009; Derks, Reijneveld, & Douw, 2014). Resting‐state functional MRI allows mapping temporally coherent brain networks, which closely resemble task‐active functional networks and depict the intrinsic neuronal organization of higher cognitive functions (Damoiseaux et al., 2006; Smith et al., 2009). Regional interactions within these resting‐state networks (RSNs), the strength of their functional connectivity, and changes thereof, have been related to the degree of cognitive impairment, assuming an optimal RSN organization to be essential for proper cognitive functioning (Bullmore & Sporns, 2012). Commonly investigated RSN in this context are the default‐mode network (DMN) and the fronto‐parietal network (FPN) (Fox & King, 2018; Lang et al., 2017; Maesawa et al., 2015). The DMN comprises cortical midline structures including the anterior cingulate cortex, precuneus, and the superior parietal lobule, and has been associated with rather task‐free processing, present as a baseline control and active in states such as mind‐wandering or self‐referential thinking (Anticevic et al., 2012; Buckner, Andrews‐Hanna, & Schacter, 2008; Raichle et al., 2001; Shen et al., 2018; Spreng, Mar, & Kim, 2009). By contrast, the FPN involves the inferior parietal lobule, inferior frontal, and inferior temporal brain regions. This network has been suggested to play a crucial role for memory and cognitive control (Cabeza & Nyberg, 2000; Higgins, Peterson, Pihl, & Lee, 2007; Kievit et al., 2014; Marek & Dosenbach, 2018; Shen et al., 2018). The two functional networks appear to be temporally anticorrelated during task performance and at rest (Anticevic et al., 2012). The strength of this dichotomy appears to be linked to the level of cognitive demand (Fox & King, 2018; Kelly, Uddin, Biswal, Castellanos, & Milham, 2008), with the degree of DMN suppression being associated with higher cognitive control (Anticevic et al., 2012).

Alterations of resting‐state functional connectivity (RSFC) within and between RSNs have been shown in healthy aging as well as in neurological and psychological disorders (Broyd et al., 2009; Jockwitz et al., 2017). Hemispheric asymmetries in RSFC have been described either as pathophysiologically relevant (Swanson et al., 2011) or as compensatory neuronal response (Cabeza, Anderson, Locantore, & McIntosh, 2002; Reuter‐Lorenz & Cappell, 2008), depending on the clinical population and functional demands. With regard to glioma patients, data is only scarce and findings appear to be inconclusive. Studies mostly found a decrease in overall DMN‐RSFC in patients as compared to control groups (Ghumman, Fortin, Noel‐Lamy, Cunnane, & Whittingstall, 2016; Maesawa et al., 2015). Tumor hemisphere and location appeared to modify DMN‐RSFC regardless of tumor size and type, indicating greater alterations in patients with left‐ as compared to right‐hemispheric (LH, RH) tumors (Ghumman et al., 2016), and linking decreased DMN integrity with diminished cognitive performance (Maesawa et al., 2015). High‐grade tumors seemed to have a higher impact on RSFC, causing largest reductions of DMN integrity (Harris et al., 2014). Regional increases in RSFC were reported as well, and DMN‐lateralization toward the contra‐tumoral hemisphere was observed in patients with low‐grade gliomas only (Esposito et al., 2012). With regard to the FPN, research evidence is rare. Lang et al. (2017) analyzed the significance of RSFC between FPN regions of interest (ROI) for presurgical cognitive performance and postsurgical cognitive outcome. They found higher overall FPN‐RSFC to be associated with poorer cognitive performance scores. Interestingly, presurgically, higher RSFC of the parietal region of the tumor‐affected hemisphere was associated with lower fluid cognition, while lower contra‐tumoral parietal RSFC was found to be predictive for worse postoperative neuropsychological outcome (Lang et al., 2017).

While previous studies partially seem to yield heterogeneous results, this might be due to content‐related and methodological differences, offering answers to different questions and limiting the comparability of findings. Notably, reported RSFC often represented whole network averages, not accounting for hemispheric differences in RSFC due to local and distant tumor effects. Although one study analyzed tumoral and nontumoral hemispheric RSFC separately, study samples included patients with LH gliomas only, but no controls (De Baene, Rutten, & Sitskoorn, 2019), so information about the potential influence of tumor hemisphere on local and distant RSFC as well as the comparison to normal is missing. However, recent studies have uncovered hemispheric asymmetries in intrinsic functional connectivity (FC) (Liu, Stufflebeam, Sepulcre, Hedden, & Buckner, 2009), implying that RSNs might vary in hemispheric vulnerability and patterns of functional reorganization depending on their neural representation (e.g., comprising predominantly midline‐ or lateral structures). In addition, RSNs may differ in their susceptibility to effects of tumor characteristics such as tumor hemisphere, location, and grade (Zhang et al., 2016). In this context, the identification of the isocitrate‐dehydrogenase (IDH) mutation (as being the most common molecular genetic alteration in Grade II and Grade III gliomas) has emerged as a major prognostic disease marker (Eckel‐Passow et al., 2015; Khan, Waqas, & Shamim, 2017). A more favorable prognosis in IDH‐mutated gliomas has been attributed to slower local tumor growth rates (Carrillo et al., 2012) and to a less infiltrative nature of diffuse tumor cell migration (Price et al., 2017). By contrast, lower structural connectivity in terms of network efficiency as well as a higher incidence of cognitive deficits have been reported for IDH‐wildtype gliomas (Kesler, Noll, Cahill, Rao, & Wefel, 2017; Wefel, Noll, Rao, & Cahill, 2016). To investigate tumor‐related asymmetries in intrinsic network architecture in relation to behavior and under consideration of prognostically relevant molecular characteristics may thus provide a better understanding of both cognitive dysfunction and neural plasticity in glioma patients.

For this reason, this prospective study investigated hemispheric RSFC differences in the DMN and FPN between patients with left‐ and right‐hemispheric gliomas (LH PAT and RH PAT), addressing tumor‐related hemispheric asymmetries in network‐specific intrinsic FC as compared to healthy controls (HC). To account for presumed tumor‐related plasticity preceding the time of disease manifestation, ICA analyses were applied to patient groups and controls separately (LH PAT, RH PAT, HC), thereby extracting (sub‐)group‐specific RSFC for each RSN. After that, contra‐ and ipsi‐tumoral hemispheric connectivity within these group‐specific RSNs were compared between groups, and the association to cognitive performance was analyzed. DMN and FPN integrity and related cognitive performance were hypothesized to be compromised in patients beyond the tumor‐affected hemisphere, and to differ depending on tumor hemisphere and investigated functional network.

## 2 METHODS

### 2.1 Participants

Thirty‐six patients with presumed cerebral glioma and 30 HC (of whom findings on microstructural white matter heterogeneity had previously been reported [Jütten et al., 2019]), were prospectively enrolled at a single university hospital center. As three patients refrained from surgery and as some of the patients were older than 75 years (for whom no age‐matched healthy control subject could be found to meet research criteria for MRI), some patients were not included in the current sample in order to ensure that sample means of patients and HC match in age. Twenty‐seven patients with histopathologically confirmed cerebral glioma (mean age: 49 ± 17 years, 15 males, 16 LH PAT, 12 IDH‐wildtype) and 27 HC (mean age: 46 ± 13 years, 16 males) were finally included in the study. Histopathological diagnoses were determined according to the revised WHO tumor classification of 2016 (Louis et al., 2016), integrating histoanatomical and moleculargenetic criteria under consideration of the IDH‐mutation status and codeletion of chromosome arms 1p and 19q in each patient.

Only patients >18 and <80 years of age with unilateral supratentorial tumors and a Karnofsky index of ≥70 were included in the study. All patients except two were naíve to tumor‐specific treatment prior to enrollment in the study. For detailed information on patients' demographics and tumor characteristics see Table 1. All participants gave informed written consent. The study was approved by the local ethics committee of the Medical Faculty of the University of the RWTH Aachen (EK294‐15) and conducted in accordance with the standards of Good Clinical Practice and the Declaration of Helsinki.

**TABLE 1:** Clinical description of included patients

Patients	IDH‐mutation	Diagnosis	Grade	Location	Side	Volume (in cm^3^)	Age (years)	Education (years)^a^	KPS
1	y	Astrocytoma	II	Temporal	r	30	30–35	13	100
2	y	Astrocytoma	II	Frontal	l	51	20–25	16	100
3	y	Astrocytoma	II	Parietal	l	64	55–60	18	80
4	y	Astrocytoma	II	Frontal	l	158	26–30	13	100
5	y	Oligodendro‐glioma	II	Frontal	r	2	36–40	13	90
6	y	Oligodendro‐glioma	II	Frontal	l	22	26–30	13	100
7	y	Anaplastic astrocytoma^b^	III	Frontal	r	30	36–40	16	90
8	y	Anaplastic astrocytoma	III	Parietal	l	114	40–45	13	80
9	y	Anaplastic astrocytoma	III	Frontal	l	49	40–45	13	90
10	y	Anaplastic astrocytoma	III	Frontal	l	21	50–55	13	90
11	y	Anaplastic astrocytoma	III	Parietal	l	119	20–25	13	90
12	y	Anaplastic astrocytoma	III	Frontal	r	155	30–35	15	90
13	y	Anaplastic astrocytoma	III	Frontal, insular	r	175	30–35	13	90
14	y	Anaplastic oligodendro‐glioma	III	Frontal	l	39	50–55	15	90
15	y	Anaplastic oligodendro‐glioma	III	Frontal	r	96	30–35	18	90
16	n	Anaplastic astrocytoma	III	Temporo‐mesial	l	51	70–75	18	70
17	n	Anaplastic oligoastrocytoma	III	Parietal	r	25	56–60	9	80
18	n	Anaplastic astrocytoma	III	Frontal	l	11	60–65	15	80
19	n	Glioblastoma multiforme	IV	Occipital	l	23	66–70	12	70
20	n	Glioblastoma multiforme	IV	Fronto‐parietal	l	11	76–80	13	70
21	n	Glioblastoma multiforme	IV	Temporal, insular	l	111	56–60	10	70
22	n	Glioblastoma multiforme	IV	Fronto‐temporal, insular	l	145	66–70	9	70
23	n	Glioblastoma multiforme	IV	Occipital	l	44	50–55	13	70
24	n	Glioblastoma multiforme^c^	IV	Temporo‐parietal	l	13	50–55	13	70
25	n	Glioblastoma multiforme	IV	Frontal	r	19	66–70	12	80
26	n	Glioblastoma multiforme	IV	Frontal	r	121	56–60	16	70
27	n	Glioblastoma multiforme	IV	Occipital	r	50	76–80	9	80

*Note*: IDH = isocitrate‐dehydrogenase, y = yes, n = no, l = left, r = right, KPS = Karnofsky performance score.

^a^ Years of education were computed by the sum of years spent for school career and further training/study.

^b^ Recurrent anaplastic astrocytoma after first tumor resection and adjuvant radiochemotherapy.

^c^ Recurrent glioblastoma after first tumor resection and adjuvant radiochemotherapy.

### 2.2 Neuropsychological assessment

All participants underwent a standardized cognitive examination, which has been described in more detail previously (Jütten et al., 2019) and included the following tests: The Verbal Learning and Memory Test (VLMT, [Helmstaedter, Lendt, & Lux, 2001]) is a list‐learning paradigm, which comprises eight trials with which verbal learning and recall are assessed. Here, we focused on VLMT scores including total learning (the sum of scores for Trials 1–5, VLMT_∑Dg1‐5) and consolidation performance as number of words forgotten over time (Trial 5 score—Trial 7 score, VLMT_Dg5‐Dg7). Maximum score is 75 indicating the best possible learning score.

The Attention Network Test (ANT) is a selective reaction time task, similar to the Posner task (Fan, McCandliss, Sommer, Raz, & Posner, 2002), that can be used to examine different attentional systems. It consists of four blocks and 288 trials, including one practice block (24 trials) as well as three test blocks (96 trials each). The participants' task is to react as quickly as possible to an arrow pointing to the right or left and to indicate that arrow's direction by button press. These arrows are imbedded in special cues or distractors intending to stimulate the various attention components. Our analyses focused on overall alertness by investigating the quotient of mean reaction times (RT) in milliseconds of correct trials and the number of correct responses (ANT_RT/Nr).

To assess executive functioning, the Trail‐Making‐Test (TMT [Reitan & Wolfson, 2004]) was carried out, which consists of two parts (TMT‐A, TMT‐B). Completion time in seconds is recorded separately for each part of the test and the difference between the two is regarded to reflect cognitive flexibility (difference in RT between TMT‐A and TMT‐B, TMT_RTexe).

### 2.3 MRI data acquisition

All participants underwent MRI examination on a 3T Siemens Prisma MRI scanner equipped with a standard 32‐channel head coil. The detailed scanning protocol is described in a previous study (Jütten et al., 2019), and comprised the following pulse sequences: A sagittal 3D T1 magnetization‐prepared rapid acquisition gradient echo (MPRAGE) sequence; a contrast‐enhanced, T1‐weighted turbo inversion recovery magnitude (TIRM) dark‐fluid sequence; a T2‐weighted TIRM dark‐fluid scan, as well as a fluid attenuation inversion recovery (FLAIR). In addition, RS‐fMRI was implemented using echo planar imaging (EPI), including 300 whole brain functional volumes, TR = 2,200 ms, TE = 30 ms, number of slices = 36 with 3.1 mm slice thickness, flip angle = 90°, and FoV = 200 mm.

### 2.4 Tumor masking

Tumor lesions were segmented semiautomatically using ITK‐SNAP software (Yushkevich et al., 2006) and included perifocal T1 hypo‐ and T2‐FLAIR hyperintensities for patients with gliomas Grade I–III, as well as T1 hypointensities and contrast‐enhancing tumor for glioblastomas. Lesion masks were inspected and manually corrected by an experienced neurosurgeon. The final masks were then used for Cost Function Masking in the following preprocessing steps and lesion volumes were computed.

### 2.5 Preprocessing and RSFC analyses

Image preprocessing was carried out using SPM12 (Friston, Ashburner, Kiebel, Nichols, & Penny, 2007; Penny, Friston, Ashburner, Kiebel, & Nichols, 2006) as implemented in Matlab 9.3 (The MathWorks, 2017). First, functional images were realigned to the mean functional volume, unwarped and coregistrated to the structural image. Structural and functional images were then normalized using the unified segmentation approach (Ashburner & Friston, 2005). In case of patients' data, a binary tumor mask was applied in the segmentation procedure to consider tumor location and size during the normalization process. Finally, functional images were smoothed with a 5 mm FWHM Gaussian kernel.

For every subject, MELODIC Single‐session ICA (Beckmann, DeLuca, Devlin, & Smith, 2005) was performed on preprocessed functional data using FSL (Jenkinson, Beckmann, Behrens, Woolrich, & Smith, 2012), applying slice‐time correction, brain extraction (BET, [Smith, 2002]) and grand‐mean scaling (mean‐based intensity normalization), generating 30 components. Of the resulting 30 components, movement related timeseries were regressed out with ICA‐AROMA (Pruim et al., 2015). MELODIC multisession temporal concatenation (Beckmann et al., 2005) was then used on high‐pass filtered data (>0.01 Hz) separately for LH PAT, RH PAT and HC. By doing so, common spatial patterns within RSN across subjects within each group were identified, accounting for possible (and depending on the affected hemisphere possibly diverse) local tumor‐dependent RSFC changes in RSN reorganization. For each of the three groups, the DMN, left and right FPN were selected as networks of interest, based on the highest correlations for spatial overlap with RSN available on FINDLAB (http://findlab.stanford.edu/functional_ROIs.html).

### 2.6 Regression analyses

Subsequently, a dual‐regression (Nickerson, Smith, Ongur, & Beckmann, 2017) analysis was applied. First, for each group, spatial maps of the DMN, left and right FPN as derived from the ICA were regressed into subject‐specific 4D datasets of each individual (spatial regression). Thereby, subject‐specific temporal dynamics of all voxels within each RSN were delineated. Next, for every subject, the extracted timeseries of each RSN were regressed into the same 4D subject‐specific dataset to identify all voxels showing a similar timeseries (temporal regression). This resulted in subject‐specific spatial maps for each RSN. Finally, one single 4D file was created for each group containing the RSN of all corresponding subjects. Each of these group 4D files was analyzed by means of a one‐sample *T*‐test using FSL's randomize tool (applying 5,000 permutations, variance‐normalization, threshold‐free cluster enhancement, and family‐wise error correction), resulting in a mean group‐specific RSN for the DMN, left and right FPN.

### 2.7 Computation of contra‐ and ipsi‐tumoral RSFC

**FIGURE 1:**
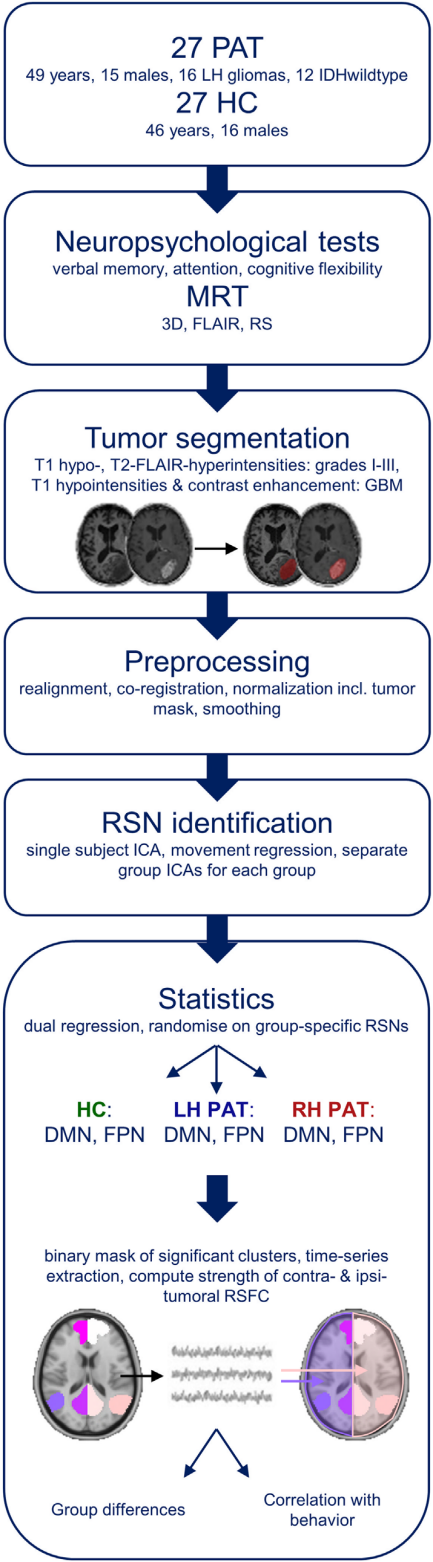
Overview of pre‐ and post‐processing steps. All glioma patients (PAT) and healthy controls (HC) underwent a standardized neuropsychological assessment as well as a structural and functional MRI. Prior to preprocessing, tumors were segmented, and binary masks were created, which were included in the preprocessing procedure of imaging data. Group‐specific resting‐state networks (RSN) were then identified for patients with left‐ and right‐hemispheric gliomas (LH PAT and RH PAT) and healthy controls (HC) separately. Those components revealing highest overlap with RSN templates available on FINDLAB (http://findlab.stanford.edu/functional_ROIs.html) were statistically analyzed, resulting in a group‐specific default‐mode‐ and fronto‐parietal network (DMN and FPN, respectively) for each of the three groups. A binary mask of significant clusters was created for each group and network, and mean timeseries of voxels within each cluster were extracted. Correlations between unilateral clusters were computed for each network in order to get an indicator of the strength of network‐specific hemispheric resting‐state functional connectivity (RSFC). Group differences in RSFC were analyzed, as well as their association with cognitive performance

To investigate differences in distant (contra‐tumoral) and local (ipsi‐) RSFC between groups, individual subjects' time‐courses were extracted within significant brain regions resulting from dual‐regression analyses described previously. To do so, significant LH and RH voxel clusters (revealing a probability threshold of *p* > .95 and extending 50 voxels) were explored for each network of interest. The identified clusters were used as ROIs, for which mean timeseries were computed and cross‐correlated per subject. Correlations were Fisher z‐transformed and hemispheric RSFC was computed. As the left and right FPN appeared to be unilateralized and comprising homolog areas in the respective contralateral hemisphere, they were combined and referred to as FPN in following analyses. LH and RH RSFC were computed for the DMN and FPN (DMN‐RSFC and FPN‐RSFC). Hemispheric DMN‐ and FPN‐RSFC differences between patients and controls were analyzed and are described in the following section. An overview summarizing all (pre‐ and post‐) processing steps is given in Figure 1.

### 2.8 Statistics

**FIGURE 2:**
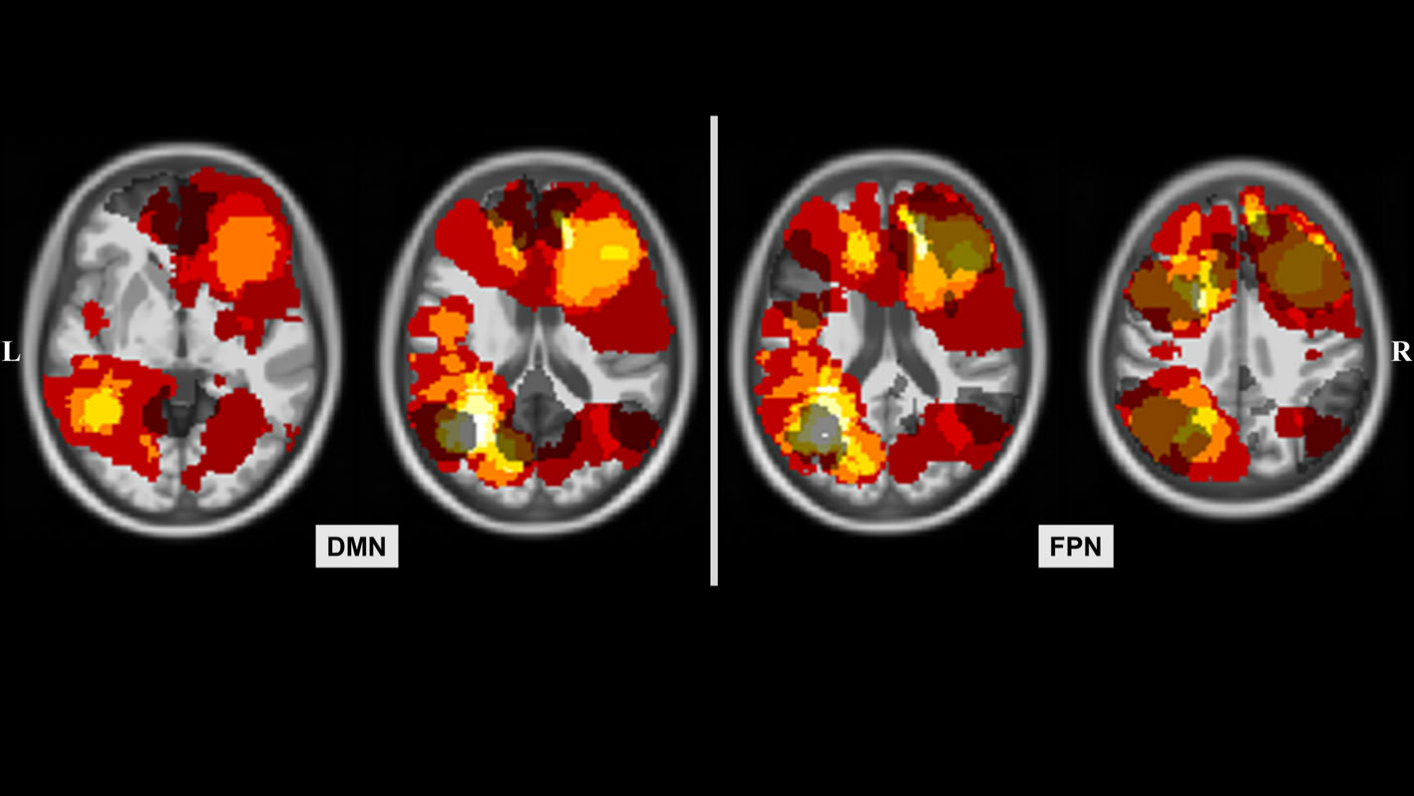
Tumor occurrence map. The distribution of different tumor locations are displayed in the neurological convention for patients with left‐ and right‐hemispheric gliomas (LH PAT and RH PAT). Regions with highest overlap in tumor occurrences are visualized in light yellow and white. The control group's default‐mode‐ and fronto‐parietal network (DMN and FPN, respectively) are overlaid in dark gray in order to visualize the overlap of patients' tumor distribution and network representations in healthy controls

All statistical analyses were performed with SPSS 24 (IBM Corp., Released, 2016). Data measures deviating more than 1.5 *SD*s from the group‐specific mean were regarded as outliers and corrected for by being replaced by the “worst” group‐specific score on that respective variable. No positive deviations (extremely good performance scores) were present. With regard to verbal learning, three performance scores were corrected (VLMT_∑Dg1‐5: HC = 3, LH PAT = 0, RH PAT = 0). Furthermore, five performance scores were adjusted for attentional performance (ANT_RT/Nr: HC = 0, LH PAT = 4, RH PAT = 1). IDH‐mutation status, tumor grade, and location (frontal, parietal, temporal, occipital, see Figure 2) were balanced between LH PAT and RH PAT (*χ* = .008, *p* = .930, *χ* = 1.350, *p* = .597, and *χ* = .926, *p* = .934, respectively). Patient groups did not differ with regard to tumor volume (*F*[1, 24] = 1.32, *p* = .262) or Karnofsky performance score (*F*[1, 24] = 1.714, *p* = .203). All statistical comparisons were tested two‐sided with a significance level of *p* < .05 and Bonferroni‐corrected for multiple testing. In addition, standardized effect sizes (ES) with the respective confidence intervals (CI, Hedges Bias corrected) were performed.

### 2.9 Cognitive performance differences

Differences in cognitive performance between patients and HC were analyzed by means of a multivariate ANCOVA, including group (patients, HC) as between‐subject factor, cognitive performance measures (VLMT_∑Dg1‐5, VLMT_Dg5‐Dg7, ANT_RT/Nr, and TMT_RTexe) as dependent variables and age as covariate. Please note that, due to physical and/or psychological strains, some patients were not able to perform all neuropsychological tests. This resulted in small LH PAT and RH PAT subgroups, which were not statistically analyzed separately, but were summarized to one group with regard to cognitive performance analyses.

### 2.10 Between‐group analyses in contra‐ and ipsi‐tumoral RSFC

In the HC, no significant difference between LH and RH RSFC was noted by the repeated measures ANCOVA, neither for DMN‐RSFC (*F*[1, 25] = 1.88, *p* = .183) nor for FPN‐RSFC (*F*[1, 25] = .02, *p* = .887). Therefore, the mean of LH and RH RSFC was computed for each network and used as baseline (“healthy”) RSFC in order to classify alterations in patients' hemispheric RSFC as being up‐ or down‐regulated.

To investigate contra ‐tumoral as well as ipsi‐tumoral effects on RSFC, hemispheric differences in RSFC between patients and HC were explored applying two multivariate ANCOVAs (for ipsi‐ and contra‐tumoral RSFC, respectively), including group (LH PAT, RH PAT, HC) as between‐subject factor, hemispheric RSFC (first: contra‐tumoral DMN and FPN, second: ipsi‐tumoral DMN, FPN) as dependent variable and age as covariate.

### 2.11 The relationship between RSFC and cognitive performance

The relationship between hemispheric RSFC and cognitive performance was analyzed for patients and HC separately using Pearson's partial correlation analyses, controlling for effects of age. As LH PAT and RH PAT subgroups were too small to be analyzed separately, patients were analyzed as one group. Correlation analyses were tested two‐sided with a significance level of *p* < .05 and Bonferroni‐corrected for multiple testing (VLMT_∑Dg1‐5, VLMT_Dg5‐Dg7, ANT_RT/Nr, TMT_RTexe, contra‐ and ipsi‐tumoral DMN‐RSFC and FPN‐RSFC; adjusted *p* = .006).

## 3 RESULTS

### 3.1 Cognitive performance differences

Cognitive performance differed significantly between patients and HC, as shown by the multivariate ANCOVA (*F*[4, 37] = 9.70, *p* < .001), revealing worse performance measures for patients with regard to verbal learning (VLMT_∑Dg1‐5: *F*[1, 40] = 28.47, *p* < .001), verbal consolidation (VLMT_Dg5‐Dg7: *F*[1, 40] = 12.78, *p* = .001), attention (ANT_RT/Nr: *F*[1, 40] = 5.92, *p* = .020) and cognitive flexibility (TMT_RTexe: *F*[1, 40] = 18.67, *p* < .001). In addition, the covariate was significant (*F*[4, 36] = 10.57, *p* < .001), revealing age‐effects on verbal learning and consolidation, attention and cognitive flexibility (VLMT_∑Dg1‐5: *F*(2, 39) = 17.80, *p* < .001; VLMT_Dg5‐Dg7: *F*(2, 39) = 10.13, *p* < .001; ANT_RT/Nr: *F*(2, 39) = 6.21, *p* = .005; and TMT_RTexe: *F*(2, 39) = 10.25, *p* < .001). For detailed results on behavioral differences, including means, effect sizes and confidence intervals, see Table 2.

**TABLE 2:** Results of group statistics on cognitive performance differences between patient groups and controls

	HC (*n* = 26)		PAT (*n* = 17)						LH PAT (*n* = 12)		RH PAT (*n* = 10)			
DV	*M*	*SE*	*M*	*SE*	*F*	*p* ^a^	ES	CI	*M*	*SE*	*M*	*SE*	ES	CI
VLMT_∑Dg1‐5	59	1.43	47	1.78	28.47	**<.001**	**−1.30**	[**−1.97** to **–0.63**]	42	3.31	44	4.81	−0.58	[−1.44–0.28]
VLMT_Dg5‐Dg7	1	0.31	3	0.39	12.78	**<.05**	0**.98**	[**.34–1.63**]	4	0.48	2	0.50	4.01	[2.56–5.46]
ANT_RT/Nr	1.9	0.05	2.1	0.06	5.92	**<.05**	0.29	[−.33–0.90]	1.9	0.03	2.4	0.27	−16.03	[−20.84 to −11.22]
TMT_RTexe	22	2.58	39	3.20	18.67	**<.001**	**1.17**	[**0.51–1.83**]	42	4.72	33	9.30	1.83	[0.84–2.83]

*Note*: DV = dependent variable, HC = healthy controls, PAT = glioma patients, LH = left‐hemispheric, RH = right‐hemispheric, *n = number* of included subjects, *M* = mean, *SE* = standard error, *F* = value of test statistic, *p* = significance, ES = effect size, CI = confidence interval, VLMT = Verbal Learning and Memory Test, ANT = Attention Network Test, TMT = Trail‐Making‐Test, VLMT_∑Dg1‐5 = sum of recalled words, VLMT_Dg5‐Dg7 = number of words forgotten, ANT_RT/Nr = quotient of reaction time and correct trials, TMT_RTexe = difference in reaction time in seconds between TMT‐A and TMT‐B.

^a^ Significant results (*p* < .05, two‐tailed), ES and CI are printed in bold.

### 3.2 Between‐group differences in contra‐ and ipsi‐tumoral RSFC

**FIGURE 3:**
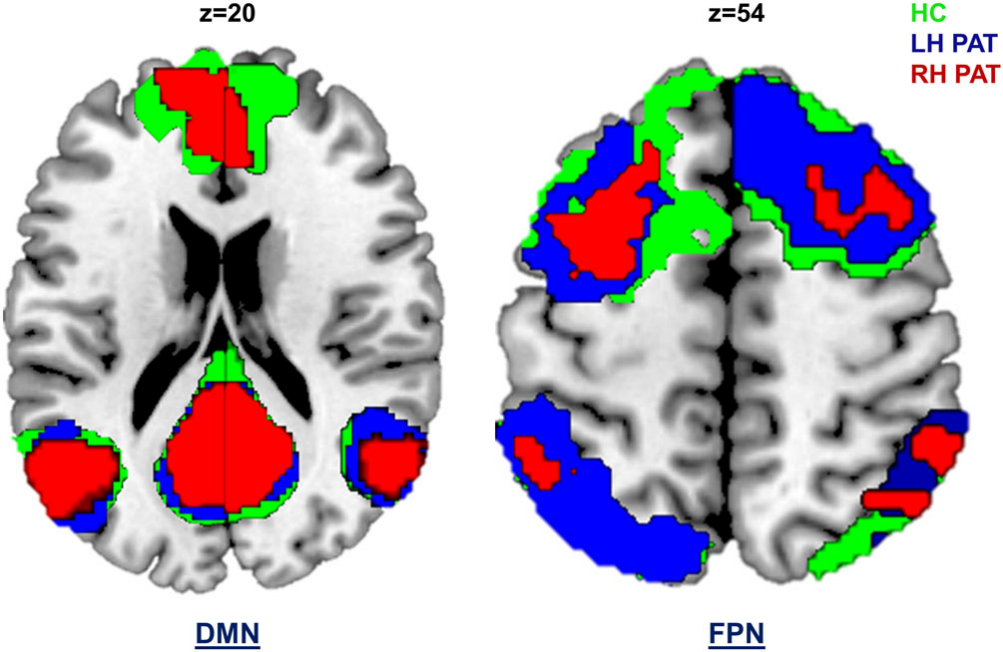
Resting‐state networks for patient groups and controls. Resting‐state networks (RSN) were computed separately for both patients with left‐ and right‐hemispheric gliomas (LH PAT and RH PAT) as well as for healthy controls (HC), and are displayed in blue, red, and green, respectively. The default‐mode network (DMN) comprised commonly reported anatomical regions, overlapping across groups in the prefrontal cortex (frontal pole and orbitofrontal cortex), the left and right inferior parietal lobule, and the posterior cingulate cortex/precuneus. Overlapping regions within the fronto‐parietal network (FPN) included the left and right intra‐parietal sulcus, parts of the left and right inferior frontal and inferior temporal gyrus

Group‐specific RSNs (DMN and FPN) are shown for patient groups and HC in Figure 3.

The multivariate ANCOVA of contra‐tumoral RSFC between patients and HC revealed significant group differences (*F*[4, 100] = 11.50, *p* < .001), both in DMN‐ and FPN‐RSFC (DMN‐RSFC: *F*(2, 50) = 17.33, *p* < .001 and FPN‐RSFC: *F*(2, 50) = 14.91, *p* < .001, respectively). Post hoc test revealed higher contra‐tumoral DMN‐RSFC in RH PAT as compared to LH PAT (*p* < .001) and HC (*p* < .001), with the latter two groups not differing significantly from each other (*p* = .158). With regard to the FPN, contra‐tumoral RSFC was higher in LH PAT and RH PAT as compared to HC (both *p* < .001) but did not differ between patient groups (*p* = 1.0). In addition, the covariate was significant (*F*[2, 49] = 3.25, *p* < .05), revealing age‐effects on FPN‐RSFC (*F*[1, 50] = 4.18, *p* < .05).

**FIGURE 4:**
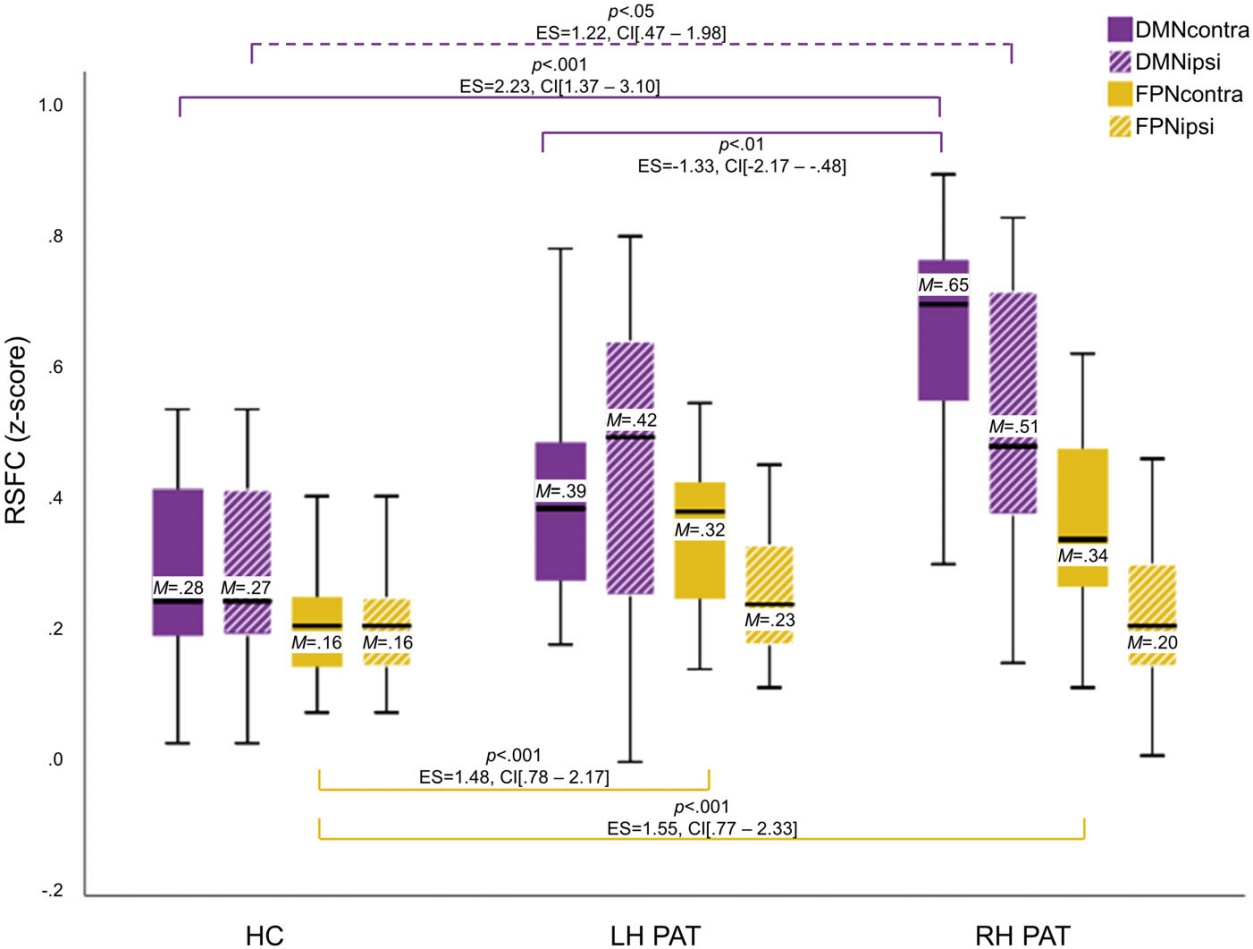
Contra‐ and ipsi‐tumoral Resting‐state functional connectivity differences between patient groups and controls. Significant differences in hemispheric resting‐state functional connectivity (RSFC) between patients with left‐ and right‐hemispheric gliomas (LH PAT and RH PAT) and healthy controls (HC) are visualized in purple for the default‐mode‐ and in yellow the fronto‐parietal network (DMN and FPN). Contra‐tumoral (filled) and ipsi‐tumoral RSFC (shaded) are displayed, including the mean (M). Significances for each analysis were computed two‐sided with a significance level of *p* < .05 and corrected for multiple comparisons, including standardized effect sizes (ES) and confidence intervals (CI)

The multivariate ANCOVA of ipsi‐tumoral RSFC revealed significant group differences between patients and HC (*F*[4, 100] = 3.00, *p* = <.05), specifically with regard to the DMN (*F*[2, 100] = 5.57, *p* < .01). Post hoc tests showed significantly higher ipsi‐tumoral DMN‐RSFC in RH PAT as compared to HC (*p* < .05). For detailed results, including means, effect sizes and confidence intervals, see Figure 4.

### 3.3 The relationship between RSFC and cognitive performance

**FIGURE 5:**
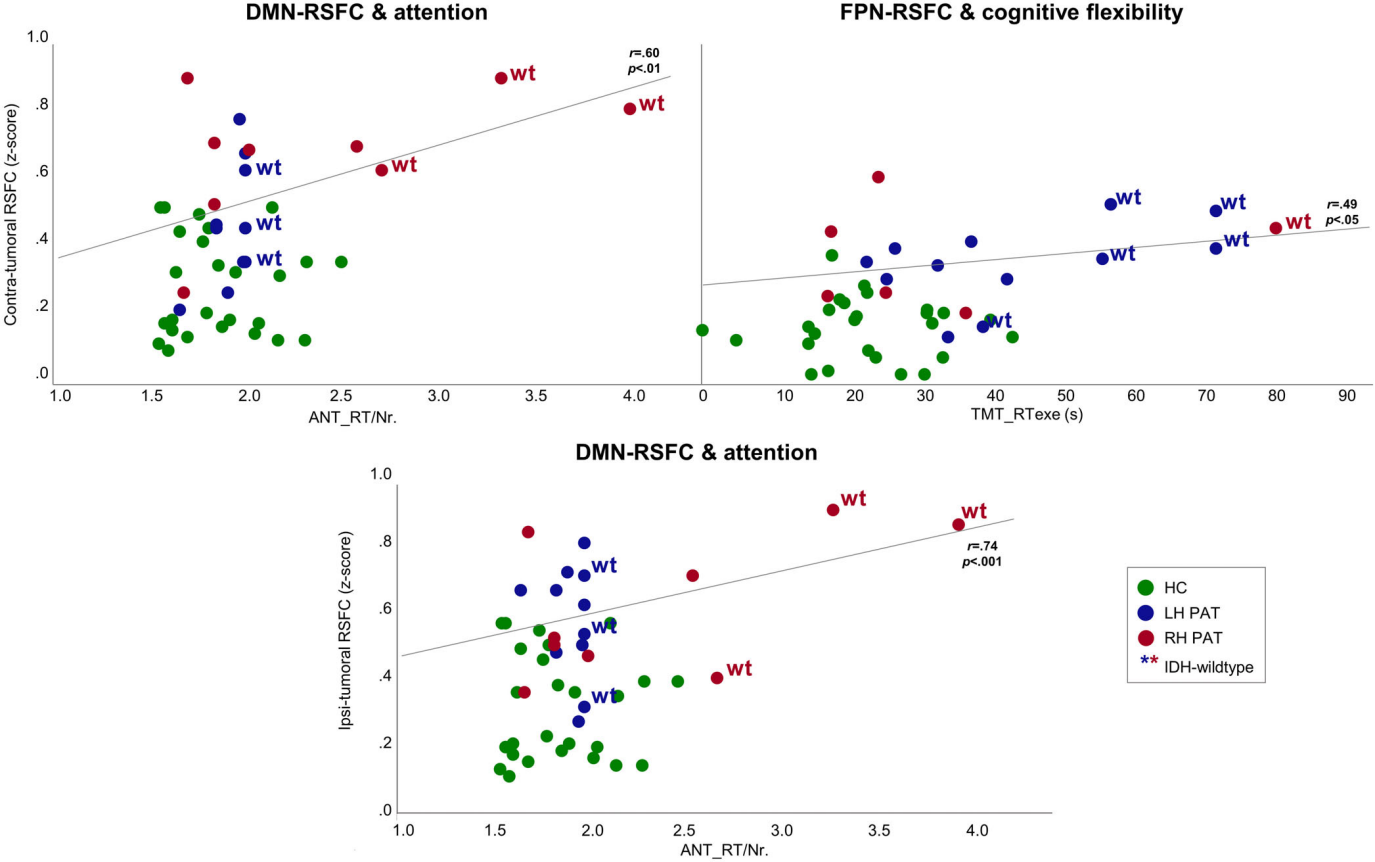
Significant associations between hemispheric Resting‐state functional connectivity and cognitive performance. Significant associations between contra‐ and ipsi‐tumoral resting‐state functional connectivity (RSFC) and cognitive performance were found in the patient group only. For reference, healthy controls (HC) are displayed in green; left‐ and right‐hemispheric glioma patients (LH PAT and RH PAT) are visualized in blue and red, respectively. Patients are further marked with regard to IDH‐mutation status, indicating IDH‐wildtype gliomas (wt) with an asterisk

Partial correlation analyses revealed significant associations between hemispheric RSFC and cognitive performance in patients. In particular, higher contra‐ as well as ipsi‐tumoral DMN‐RSFC was associated with worse attention performance (DMN‐RSFC_CONTRA_‐ANT_RT/Nr: *r*_partial_ = .60, *p* < .01; DMN‐RSFC_IPSI_‐ANT_RT/Nr: *r*_partial_ = .74, *p* < .001). In addition, higher contra‐tumoral FPN‐RSFC was associated with worse performance regarding cognitive flexibility (FPN‐RSFC_CONTRA_‐TMT_RTexe: *r*_partial_ = .49, *p* < .05). After Bonferroni‐correction for multiple testing, however, only the association between ipsi‐tumoral DMN‐RSFC and attention performance remained significant at the adjusted *p*‐level (*p* = .006). Plotting these associations between contra‐tumoral RSFC and cognitive performance revealed individual outliers. Those were inspected in more detail with regard to IDH‐mutation status and tumor location. As IDH‐mutation status and/or tumor location could not be analyzed with inferential statistics due to too small subgroups, tumor characteristics were inspected on an explorative basis only. While tumor location varied, all but one of these outliers represented IDH‐wildtype gliomas (see Figure 5). For the HC, correlations did not reach significance.

## 4 DISCUSSION

This prospective study investigated hemispheric RSFC differences in the DMN and FPN between LH PAT and RH PAT, addressing effects of asymmetry the tumor might have on network‐specific intrinsic FC. Hemispheric RSFC in patients varied depending on tumor hemisphere and investigated network, showing a dissociation between DMN‐ and FPN‐RSFC alterations. While both patient groups showed FPN‐RSFC increases in the respective contra‐tumoral hemisphere, DMN‐RSFC was increased in the dominant left hemisphere, irrespective of tumor side, and with most prominent alterations being found in RH PAT. However, RSFC increases did not necessarily appear to be beneficial for performance in terms of better performance measures, but to vary depending on tumor hemisphere, cognitive demand and seemingly on the prognostically relevant IDH‐mutation status, which will be discussed in detail below.

### 4.1 Cognitive performance and glioma‐associated DMN‐ and FPN‐RSFC asymmetries

In line with previous research, patients showed compromises in cognitive performance, revealing worse performance measures than controls in all cognitive domains tested, and with most distinctive deficits in verbal memory. Cognitive dysfunction has been reported to occur already early in the disease course, and often prior to other tumor‐related symptoms (Habets et al., 2014; Van Kessel et al., 2017). Moreover, deficits arise across multiple domains (De Baene et al., 2019) and can hardly be explained by tumor‐induced local disruptions alone, but rather suggest long‐range effects with lesion impact on underlying network‐based functional (re‐)organization (Bullmore & Sporns, 2009). Complying with this notion, we found increased hemispheric DMN‐ and FPN‐RSFC in patients across both hemispheres and RSNs. However, differences between hemispheres showed a dissociation depending on tumor side and investigated RSN: While LH PAT and RH PAT showed higher FPN‐RSFC increases in the respective contra‐tumoral hemisphere, hemispheric DMN‐RSFC was increased in the dominant left hemisphere in both patient groups, irrespective of tumor side, albeit with highest RSFC increases in RH PAT. With regard to FPN‐RSFC alterations in glioma patients, research evidence is rare. To our knowledge, Lang et al. (2017) and De Baene et al. (2019) were the only ones to investigate tumor‐related RSFC alterations and network integrity of the FPN with regard to the relevance of cognitive performance. However, studies contrasting hemispheric FPN‐RSFC alterations between patients and HC or between LH and RH PAT are completely lacking, making it difficult to discuss the results in light of the literature. By contrast, RSFC within the DMN has been investigated more extensively. While several previous studies reported tumor‐related decreases in DMN‐RSFC (Esposito et al., 2012; Ghumman et al., 2016; Maesawa et al., 2015), regional increases of RSFC within the DMN have been described as well, with lateralization toward the contra‐tumoral hemisphere in low grade glioma patients (Esposito et al., 2012). In addition, a difference in RSN vulnerability to tumor‐related effects has been suggested (Fox & King, 2018), which is supported by recent findings. For example, Zhang et al. (2016) investigated alterations of the posterior DMN in LH and RH frontal gliomas and found a decrease in strength of hemispheric FC within the left hemisphere for both patient groups, suggesting a higher vulnerability to glioma‐related influences of the dominant hemisphere (Zhang et al., 2016). Our results agree with these findings in that the tumor had asymmetric effects on the DMN. Seemingly diverging, we found an increase of hemispheric DMN‐RSFC in the dominant hemisphere, with most pronounced alterations in RH PAT. Notably, taking into account the potential tumor‐related spatiotemporal reallocation of RSNs, hemispheric means of RSFC in our study also comprised other than the core network brain regions alone (nonhubs and hubs, respectively), while Zhang et al. (2016) investigated hub–hub connectivity only. In this context, alterations of DMN and FPN connectivity profiles in glioma patients have recently been described by Derks et al. (2017), who defined a complex functional connectome based on a whole‐brain gray matter parcellation with empirically defined hubs and nonhubs. Results indicated a higher connectivity over all connectomic links in patients as compared to HC, with lower between‐hub connectivity, but increased hub to nonhub connectivity. Connectomic profiles further varied depending on tumor grade, with most distinct alterations in Grade III gliomas as compared to Grade II and IV tumors.

While anatomically defined nodes allow characterizing between‐hub connectivity within empirically defined RSNs, this may not sufficiently account for tumor‐induced plasticity with potential spatiotemporal reallocation of RSNs and hubs. Thus, applying data‐driven voxel‐based analyses may for example, delineate the involvement of additional brain regions (or the lack of regions normally involved) in the context of tumor‐related RSN reorganization. Moreover, in view of the revised WHO classification criteria (Louis et al., 2016) with moleculargenetic criteria having emerged as major prognostic disease marker (Eckel‐Passow et al., 2015), FC profiles should be reassessed under consideration of prognostically differing genotypes.

Overall, the comparability of previous research findings seems limited due to the heterogeneity of study cohorts and due to methodological differences. Some studies did not include a (demographically matched) control group (De Baene et al., 2019; Lang et al., 2017), so RSFC was described as higher or lower relative to the contra‐lesional hemisphere or to other patients, but not relative to HC. Other studies included previously treated patients (Harris et al., 2014), or different tumor entities such as noninfiltrating extra‐axial tumors (Ghumman et al., 2016). Only few acquired behavioral data in order to correlate RSFC alterations with cognitive performance (De Baene et al., 2019; Lang et al., 2017; Maesawa et al., 2015). Thus, results should be interpreted carefully depending on the study cohort and methodological approach applied, but they do not necessarily represent contradictory conditions. Rather, different tumor‐related adaptive processes may as well occur in parallel or at different disease stages, and might further be influenced by tumor growth dynamics.

### 4.2 Association between RSFC and cognitive performance

Altered FC as a sign of RSN disintegrity and associated cognitive decline have been described in previous research. Specifically, decreases in RSFC have been linked to cognitive dysfunction in tumor patients (Ghumman et al., 2016; Maesawa et al., 2015), and RSN disintegration has been described to be more pronounced in high as compared to low grade glioma patients (Harris et al., 2014). Complying with previous these findings, cognitive performance in our patients was associated with RSFC alterations. The present study found increases in hemispheric DMN‐ and FPN‐RSFC to be associated with worse cognitive performance, which is in line with previous research linking increased FPN‐RSFC and lower cognitive scores in glioma patients (Lang et al., 2017). While results might initially be interpreted as tumor‐related loss of functional efficiency, plotting the associations between RSFC and cognitive performance revealed individual outliers (Figure 5). On an exploratory basis, these were inspected in more detail with regard to different tumor characteristics. Interestingly, all but one of these patients represented IDH‐wildtype gliomas, while varying in tumor location. When disregarding these patients, increased RSFC measures in the remaining patients appeared to be accompanied by cognitive performance levels comparable to those of the HC. Based on inspecting the correlation plots, mean hemispheric RSFC between patients with or without IDH‐mutation status did not seem to differ, while cognitive performance appeared to be worse in patients with IDH‐wildtype gliomas. This was particularly the case if tumors were located in the hemisphere associated with the corresponding cognitive function. Accordingly, IDH‐wildtype RH PAT performed worse than IDH‐mutated RH PAT and all LH PAT on the attention task. The equivalent pattern was found for IDH‐wildtype LH PAT with regard to cognitive flexibility. It might thus be suggested that this variability in RSFC alterations relate to compensatory processes, depending on cognitive demand and affected hemisphere, but which may vary in efficiency depending on IDH‐mutation status. While increases in RSFC in patients with IDH‐mutated gliomas might be compensatory, RSFC increases seemed to be insufficient in patients with IDH‐wildtype gliomas, who reflect the prognostically less favorable and more rapidly progressing tumor type. A higher functional reserve capacity in less aggressive tumor types seems plausible considering that chronically progressive brain lesions may lead to focal disruptions, but allow neural plasticity to accompany the disease process, which appears more likely the slower the tumor growth. This idea is supported by Kesler et al. (2017), who investigated the role of IDH‐mutation status for structural connectivity and cognition. Authors found lower network efficiency and a higher incidence of cognitive deficits in IDH‐wildtype gliomas. Network efficiency and cognitive impairment were inversely associated in both IDH groups, and cognitive reserve capacity appeared to mediate this effect in IDH‐mutated gliomas (Kesler et al., 2017). Increases in FC with recruitment of additional or alternate brain regions or functional lateralization toward the unaffected hemisphere might therefore represent attempts of functional compensation. If, however, the velocity of tumor proliferation overrides compensatory neural reorganization processes, functional reserve capacities might be exhausted, resulting in behavioral dysfunction. Nevertheless, due to the small sample size of patient subgroups and resulting limitations regarding the statistical analysis of the data, these findings require replication and confirmation.

### 4.3 Limitations and future perspectives

The small sample size in our cohort limited the interpretability of results and impeded further subanalyses of the potential impact of different tumor characteristics on RSFC. Specifically, behavioral and RSFC group differences between patients with and without IDH mutation status could not be objectified statistically. This could however be of interest for future studies in order to gain insights into neural correlates of cognitive functions and neural plasticity of prognostically heterogeneous patient groups. In this context, future studies may further focus on phenotyping different prognostically relevant moleculargenetic profiles including the IDH mutation status, codeletion of chromosomes 1p/19q, or the TERT promoter mutation (Eckel‐Passow et al., 2015), which was beyond the scope of the present study. Similarly, the small sample size did not allow analyzing effects of different tumor locations on RSFC and behavior. It can be assumed that different tumor locations impact differently on intrinsic network architecture, depending, for example, on whether or not network‐relevant anatomical regions (hubs) are affected by the tumor lesion. Interestingly, even though RH and LH tumor distributions may not have been mirrored perfectly in our cohort, LH DMN‐RSFC was found to be increased (at least relative to the right hemisphere and relative to HC) both in RH and LH PAT, while FPN‐RSFC was increased in the respective contra‐tumoral hemisphere, independent of tumor side. This observation of a dissociation between tumor‐related DMN‐ and FPN‐RSFC alterations in our cohort may be challenged when larger cohorts with different tumor locations, ‐sizes and ‐growth characteristics will be analyzed, as RSN vulnerability and ‐plasticity may depend on multiple factors. Thus, future studies with larger and more homogeneous samples are desirable to allow a more systematic approach to the current subject. Nonetheless, the focus of this study was the comparison of hemispheric RSFC alterations between LH and RH PAT, thereby addressing effects of asymmetry the tumor might have on intrinsic FC. As IDH‐mutation status and tumor locations were balanced between patient groups, group differences in RSFC due to these factors are at least unlikely. The present results indicate lesion‐induced global RSFC alterations, extending from local changes to the contra‐tumoral, seemingly nonaffected hemisphere. Effect sizes do support results of the test statistics, hence suggesting an impairment of RSFC beyond local tumor effects, and agree with current concepts of a network rather than modular based organization of higher brain functions. Phenotyping RSN‐specific connectivity profiles in glioma patients may allow a more suitable estimate of cognitive outcome in surgical interventions and might further provide an additional diagnostic and prognostic marker, aiding in treatment monitoring.

Another limitation of the study is that patients and HC differed significantly in years of education (*p* < .001), which did not allow including educational level as covariate in our analyses. On average, patients had 2.5 years less educational years than the control group. However, all patients had achieved at least secondary school level. With this common level of education, we considered an educational difference of 2.5 years as at least unlikely to affect performance in the kind of tests applied.

A further limitation is the fact that neurovascular uncoupling occurs in tumor patients, which is known to impact the Blood‐Oxygenation Level Dependent (BOLD) signal by tumor related changes of the neurovasculature and brain perfusion (Sun et al., 2020). Thus, it should be considered that RSFC alterations in patients might be an expression of tumor‐related alterations of brain perfusion and neurovascular uncoupling. Future studies elucidating the relation of perfusion and BOLD signal changes in glioma patients are needed to provide a better insight into the true physiological basis of presumed RSFC measures.

## 5 CONCLUSIONS

Hemispheric RSFC in glioma patients varied depending on tumor hemisphere and investigated network, indicating a dissociation between tumor‐related DMN‐ and FPN‐RSFC changes. Hemispheric RSFC increases were not necessarily found to be beneficial for performance in terms of better performance measures in patients, but to vary depending on tumor side and cognitive demand, which seemed to be further altered by IDH‐mutation status. Characterization of tumor‐related asymmetries in intrinsic network architecture may provide a better understanding of both cognitive dysfunction and neural plasticity in glioma patients. Future studies should further elucidate the influence of different tumor characteristics and prognostically different moleculargenetic profiles on intrinsic network dynamics.

## Data Availability

The data that support the findings of this study are available on request from the corresponding author. The data are not publicly available due to privacy or ethical restrictions.
